# Calculating cumulative effects in GIS using a stepless multivariate model

**DOI:** 10.1016/j.mex.2021.101407

**Published:** 2021-06-06

**Authors:** L. Erikstad, V. Bakkestuen

**Affiliations:** Norwegian Institute for Nature Research, Norway

**Keywords:** Environmental impact assessment (EIA), Cumulative effects, Multivariate Analysis, Principal Component Analysis (PCA), GIS, Overlay analysis

## Abstract

The paper present a streamlined workflow, using multivariate analyses of environmental variables in combinations with GIS overlay analyses that provide methods to extract and analyse major environmental and climatic gradients by using fishnet polygons as sample units. The method opens for illustrating multivariate results as geographical maps and as PCA plots using sample scores as coordinates. Then the PCA sample scores can be allocated to fishnets polygons and each sample score can be assigned with its ID and other attributes to each fishnet polygon. This is used to construct a cumulative impact model based on PCA fishnet polygon frequency scores and further to measure representativity of nature protected areas. It also provide possibilities for testing of a range of different hypothesis. The method present the numerical results visually in both the PCA sample score plot and in a geographical map, and can be used as a part of cumulative impact analysis to assess representativeness of mapped or modelled valued environmental components (VECs). It can be applied to existing as well as planned or potential infrastructure and other technical developments.

• The Stepless Multivariate Model is an explicit, transferable and reproducible procedure to conduct systematic assessment of cumulative impacts based on an analysis of representativity.
• The method can be used to illustrate the analysis both geographically and numerically.
• The procedure in the method has a potential wide range of applications and can form a basis for hypothesis testing.

## Graphical abstract

**Figure:**
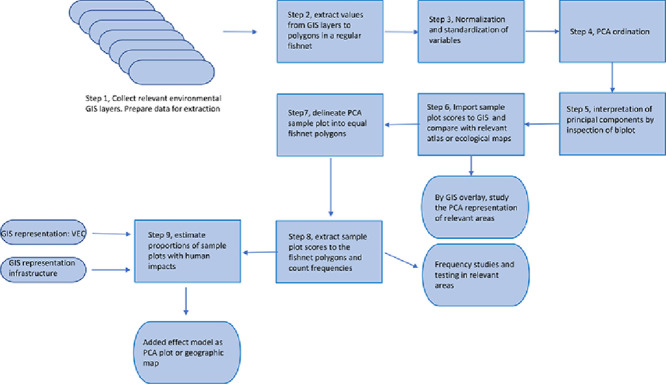

Specifications table

**Table:** 

Subject Area:	Environmental Science
More specific subject area:	Numerical GIS analysis of environmental data for planning
Method name:	Utilizing the interface between PCA plots and GIS overlay analyses
Name and reference of original method:	The original method is referred to as “PCA-Norway” among Norwegian nature research scientists and by the management, and published in Bakkestuen, V., Erikstad, L. & Økland, R.H. 2008. Step-less models for regional environmental variation in Norway. – J. Biogeography 35: 1906-1922.
Resource availability:	The sources of data were: terrain data (100-m resolution digital elevation model, DEM) from the Norwegian Mapping Authorities; raster climatic data with 1-km resolution based on the 1960–90 normal ([2]a; [7]) compiled by the Meteorological Institute ([15], 2000); hydrological data from the Norwegian Water Resources and Energy Directorate; and geological data from the Norwegian Geological Survey (based on vector data scale 1:250,000) For resources elsewhere (than Norway): National databases. Supplements: Climate data are widely available through WorldClim [8], Google Earth Engine (GEE) or national databases. Terrain or elevations models (DTMs or DEMs) are available from HYDRO1k, from SRTM or MERIT DEM which is available in GEE, and national databases. Similar terrain derivative variables as used in [3] or other relevant ecological terrain variables, can be derived from elevation models as described in Amatulli et al. [1]. Other sources that are globally available, are land use maps and satellite derived outputs, from for instance from the COPERNICUS NASA and ESA programs. These are freely accessible in GEE or in national data servers. Many countries also have their own ecological base maps that often can be accessible in finer or more detailed scales. For open access GIS we can recommend programs or servers like QGIS, GDAL, GRASS or Google Earth Engine.

The method described consist of two main elements:

• Establishing a step-less model for regional environmental variation in the region of interest and.
• Using this model to assess representativity and cumulative effects.

The first element is previously presented in Bakkestuen et.al. (2008) [3]. This procedure for establishing the stepless model has three main stages: 1- Data acquisition, 2- Data preparation and ordination and 3- Interpretation. The second element that handles representativity and cumulative effects are based on methods in the associated paper Erikstad et al. [5].

## Tutorial

**Fig. 1:**
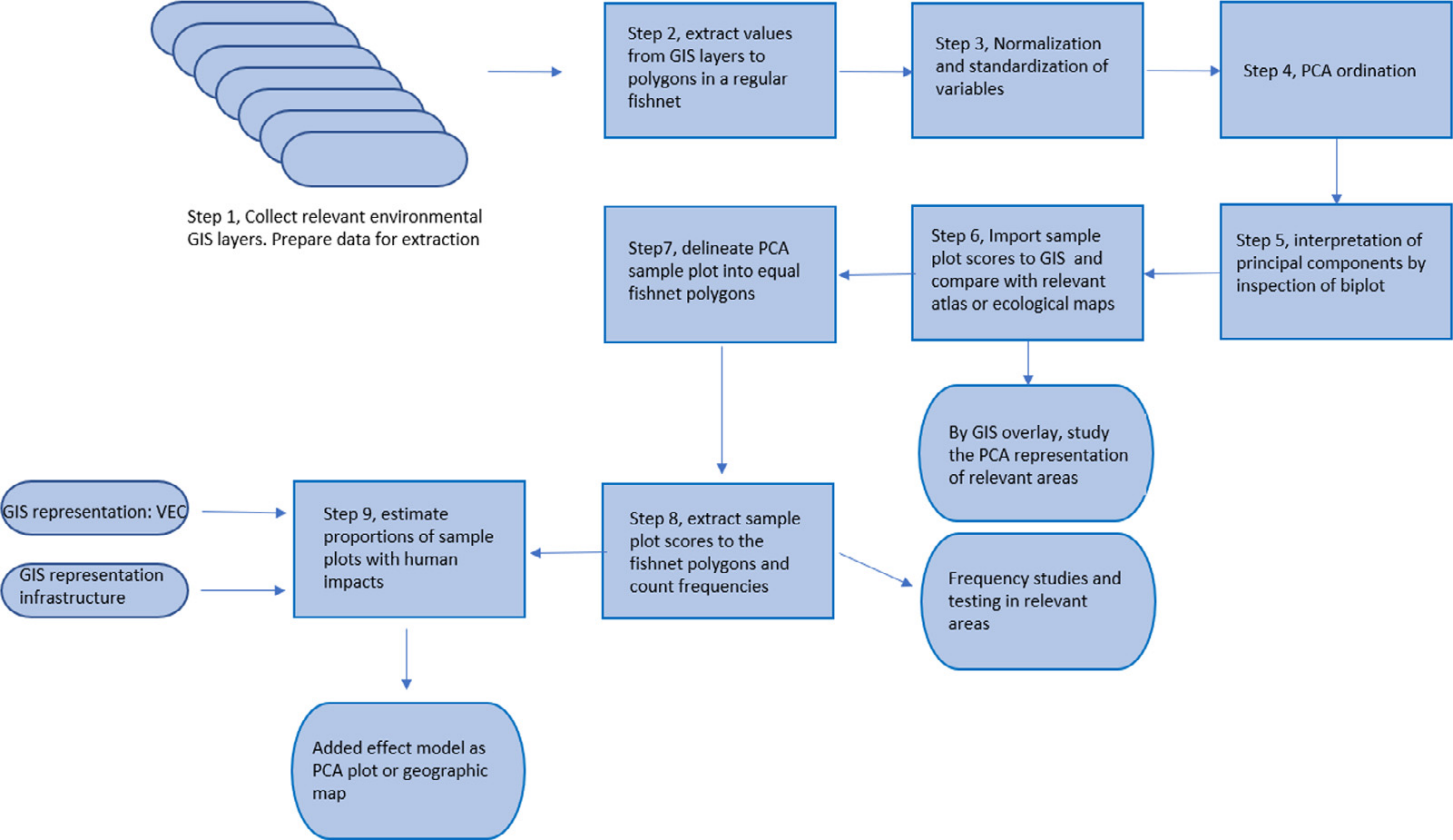
Graphical overview of the method presented.

This tutorial includes nine separates steps. These are summarized in Fig. 1.

## Data acquisition

(**Step 1**) Available GIS layers that are assumed to be of importance for environmental variation and gradients in the targeted area should be gathered in a GIS project and stacked upon each other. This can be done in a freely available desktop GIS like QGIS or similar software. The selection of GIS layers should focus on those that are believed to contribute in explaining the main environmental variation at the spatial extent at your targeted area. As an example, a large targeted area should contain climatical data and a small targeted area should contain high resolution topographic data or maybe soil characteristics etc.

(**Step 2**) Here you superimpose a fishnet of desired size (extent and resolution) on the environmental layers and extract values to the fishnet polygons. You will now extract data from your GIS layers to the each of the polygons in the fishnet. This procedure will have different names depending on your GIS software; it is called zonal statistics in QGIS, zonal statistics as table in ArcMap, and ArcGIS Pro and reduce regions in Google Earth Engine to name some commonly used software. You can summarize or extract (the terms used synonymously here) your data by different measures or reducers like sum, max, min, range, variance etc.

## Data preparation

(**Step 3**) Normalization and standardization of variables

All variables should be transformed to zero-skewness and kurtosis standardized by division with their expected standard deviations, (6/n)^0.5^ [14]. Acceptable homogeneity of variances (homoscedasticity) is achieved by transforming all variables to zero skewness (using transformation formulae of [11]):

Three transformation formulae according to Økland et al. [11] can be used:

(1) $${\text{ykj}}^{\prime }=\text{eckxkj}$$

(2) $${\text{ykj}}^{\prime }=\text{ln}\left(\text{ck}+\text{xkj}\right)$$

(3) $${\text{ykj}}^{\prime }=\text{ln}\left(\text{ck}+\text{ln}\left(\text{ck}+\text{xkj}\right)\right)$$

where xkj is the original value of variable k in plot j and ck is a variable specific parameter that gives the transformed variable Y´ = {ykj´} zero skewness. The first equation is applied to left-skewed variables (standardized skewness < 0), the next equation to right-skewed variables. The last equation is applied to right-skewed for which no ck could be found by the middle equation that resulted in standardised skewness = 0. After transformation, all variables Y´ were ranged to obtain new variables Y = {ykj} on a 0-1 scale:

(4) $$\text{ykj}=\left({\text{ykj}}^{\prime }-\text{min}\left({\text{ykj}}^{\prime }\right)\right)/\left(\text{max}\left(\text{ykj}\right)-\text{min}\left(\text{ykj}\right)\right)$$

## Ordination and interpretations

**Fig. 2:**
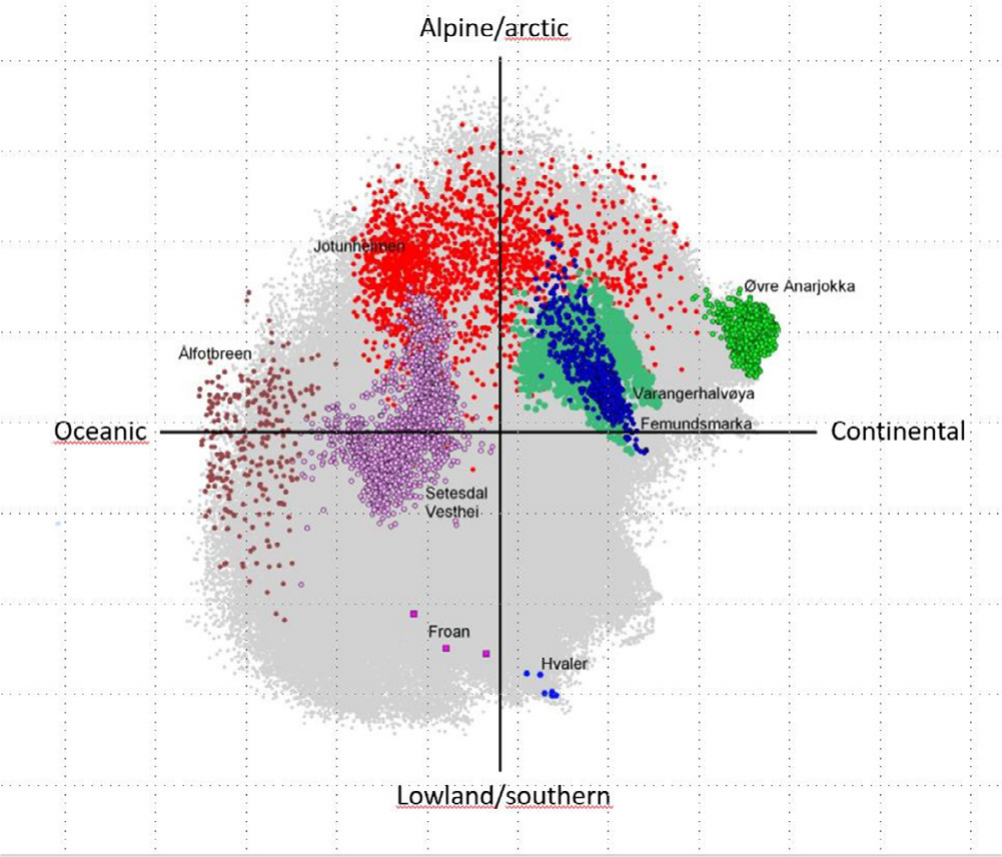
The representation of eight National Parks in the PCA sample plot in a GIS project, indicating how much of the bioclimatic variation in Norway that is captured by these areas.

(**Step 4**) The standardized and normalized matrix is now ready to be subjected to PCA ordination [4]. PCA ordination can be performed by using the vegan package [12] in R software (R Development Core Team 2021). The same procedure are available in open access GIS programs like QGIS and Google Earth Engine.

(**Step 5**) Interpretation of PCA and principal components is based on variation explained by components and finding which (“species” or environmental) variables are most strongly correlated with each principal component. By producing a biplot, these correlations can be interpreted visually. Variables most closely correlated to each component are the ones with highest loadings along the component axes. Accordingly, the ones with low loadings are little correlated with the corresponding component. Loadings along principal components makes a vector that show which directions sample variable values increases. In a two-dimensional biplot, the sample plots that are positioned in the same direction as the environmental vectors generally contain increasing values for this environmental variable . For instance, sample plots that are located in the same direction as the elevation vector are the ones most closely correlated, i.e. the elevation will increase in sample plots along the direction of the elevation vector.

(**Step 6**) The sample plot scores from the PCA should now be imported to the GIS as an attribute table. Now you shall make two GIS projects or one project with two views showing (1) the PCA diagram by adding sample plot scores from the two first principal components (or one or two others) as x and y to the GIS normally performed by a procedure called “add X and Y point data” and (2) join the fishnet polygons with principal components values into the fishnet polygon attribute table. The PCA sample plot scores can then be visualized in the GIS PCA diagram and geographically on the fishnet. The resulting patterns can be assessed and further interpreted in combination with other geographical maps like existing botanical atlas maps or other ecological relevant maps. Note that PCA-scores are often centered around zero and contain both positive or negative values, which may switch between being positive and negative when running PCA several times. An example of having a PCA diagram in a GIS project is shown in Fig. 2.

(**Step 7**) Step 7 is to divide the PCA sample plot score diagram into PCA diagram fishnet polygons. The sizes of these fishnet polygons can be chosen independently for each study regarding how much details that you want this method to segregate. PCA sample plot scores are normally standardized from values −1 to 1. We have experienced that dividing the PCA diagram fishnet into 20 equally large segments is sufficient for assessing cumulative effects. Each segment polygon thus represents an approximate grouping of environmental conditions in similar segments, It is important to notice that this grouping per definition is environmental and not geographical. The division of the PCA sample plot makes it possible to study the frequency of samples in each sample plot segment (fishnet polygons) for a more detailed analysis of the PCA diagram when the diagram consist of an overwhelming number of samples.

(**Step 8**) Step 8 is a process similar to step 2 where the frequencies of occurrences of sample plots are counted in the PCA diagram fishnet polygons. Having access to the sample attribute table makes it possible to visualize how much ecological or bioclimatical space each attribute span in the statistical PCA model. The attributes may represent a county or for instance different national parks (Fig. 1) or an area with defined environmental characteristics or value (valued environmental component – VEC). This is a visual step towards an assessment of representativeness.

(**Step 9**) Step 9 consists of calculating the proportions of sample plots with human impact. Here occurrences of human impacts in each environmental segment related with the VECs are counted and summed up. The proportions of influenced polygons in each ecological segments is now an indicator of added or cumulative effects.

An extra optional step is to import the indicator of accumulated effect values back to the GIS to visualize these geographically as part of a map of the area of interest as a point file or aggregated in a relevant polygon file.

## A walk through example based on real data

The data set used in this walk through was first reported in Bakkestuen et.al. 2008 [3] at a resolution of 5 km pixel size for all mainland Norway. It was further developed and presented in an associated paper by Erikstad et al. [5] in a more detailed resolution (1km pixel size as well as a courser size of 10 km). It contains fifty-four climatic, topographical, hydrological and geological variables. These variables was collected from different types of data sources. A full list of variables of all types are listed and explained in Table 1 in Bakkestuen et al. [3].

All variables were summarized in ArcGIS Spatial Analyst as grid-cell means with the exception of elevation relief (which was recorded as the range of observed elevation values in each cell) and terrain variation (standard deviation of observed slopes for the grid cell). The procedure followed the tutorial described in this article.

**Fig. 3:**
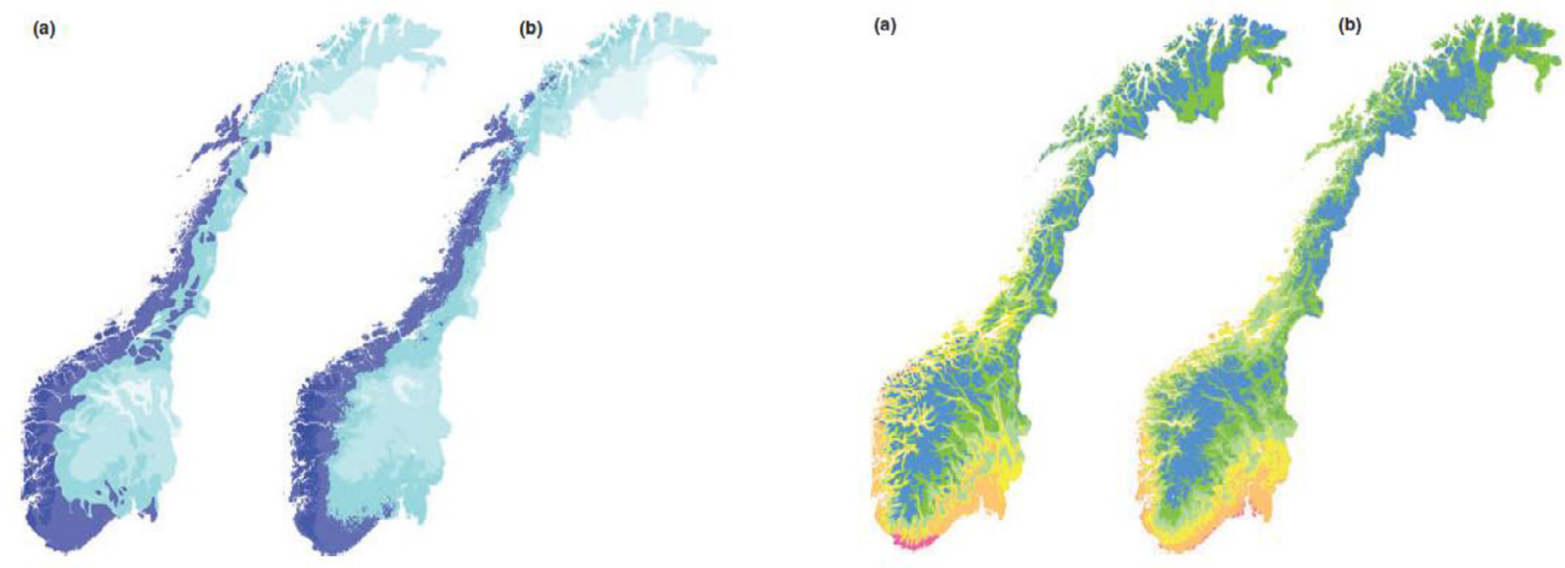
The two first PCA gradients. The first interpreted as a oceanic to continental gradient and the second a temperature gradient going from lowland/southern latitudes to highland and arctic condition. Figure marked a was the result of the PCA, while b is the original mapping of vegetation zones and sections by [10].

The first four principal components explained between 75% and 85% of the variation in the data sets dependent on the resolution between1 × 1 km and 10 × 10 km sample plot sizes. The PCAs revealed four consistent environmental gradients, in order of decreasing importance: (1) regional variation (gradient) from coast to inland and from oceanic/humid to continental areas; (2) regional variation from north to south and from high to low altitudes; (3) regional variation from north to south and from inland to coast, related to solar radiation; and (4) topographic (terrain relief) variation on finer scales than (1–3). The first two PCA axes corresponded to the two bioclimatic gradients used in expert classifications of Norway into biogeographical regions: vegetation sections (from highly oceanic to slightly continental) and vegetation zones (from nemoral to alpine zones). A comparison between PCA ordination results and the expert classification Fig 3 into vegetation regions by Moen [10], represented as a map scaled 1:1,000,000, was made by Bakkestuen et al. [3] in several steps. First, the area covered by each vegetation zone and section [10] was calculated by using the option for summarizing zones in ArcView Spatial Analyst. Second, 1-km grid cells that were homogeneous, in the sense that they were assigned uniquely to one of Moen's zones or one of the sections, were assigned to this class, while other cells were discarded from further analysis. Third, we calculated the position of each grid cell along each of 360 directions (adjacent directions separated by an angle of 1) in the space defined by PCA ordination axes 1 and 2. This was done because the best correspondence between PCA positions and Moen's zones, and sections and zones, respectively, might not be along the main PCA axes themselves, but along combinations of axes. For each direction, the rank-ordered homogeneous grid-cell positions were divided into groups, so that the number of cells in each group was proportional to the relative area of the zones, ordered from nemoral to alpine zones and vice versa, and with the relative areas of the sections, ordered from strongly oceanic to slightly continental, and vice versa. Fourth, for each of Moen's zones and sections, we found the directions with the highest grid-cell concordance (the highest fraction of homogeneous grid cells correctly classified to zone or section). These directions are termed the step-less zone model and the step-less section model, respectively.

**Fig. 4:**
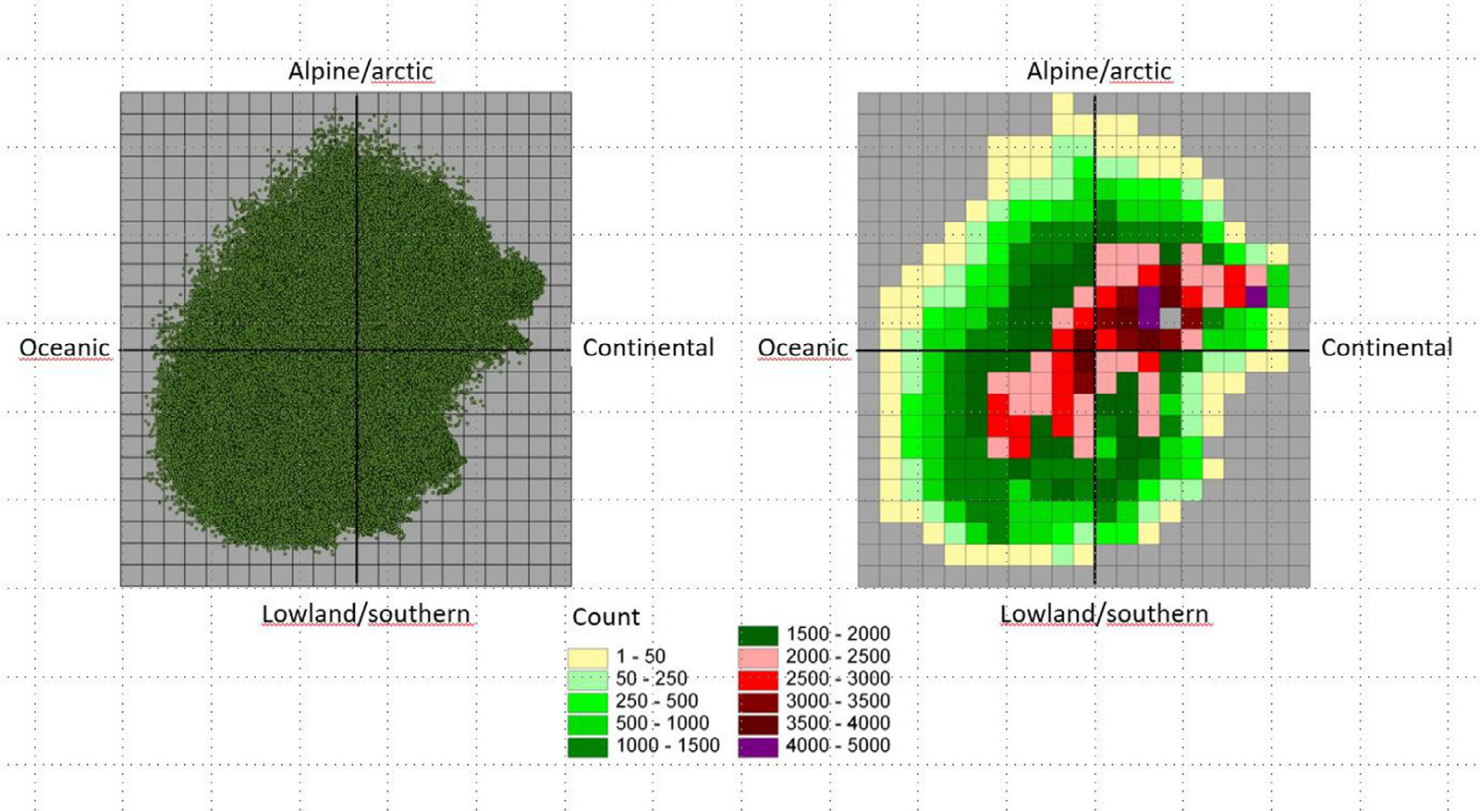
To the left is the PCA sample plot diagram for Norway based on the two first PCA axes in Bakkestuen et al. [3]. To the right frequency of sample scores based on a 1/20 of the gradient length are shown. Note that even if the plots are spread neatly over a large area over the diagram, the frequency reveals a gravity of the Norwegian land mass more in the direction of arctic and continental conditions.

We divided each segment to represent 1/20 of the gradient spanned by the PCA-axis for the whole national model (Fig. 4) and the PCA representation of the given VEC was calculated and visualized.

**Fig. 5:**
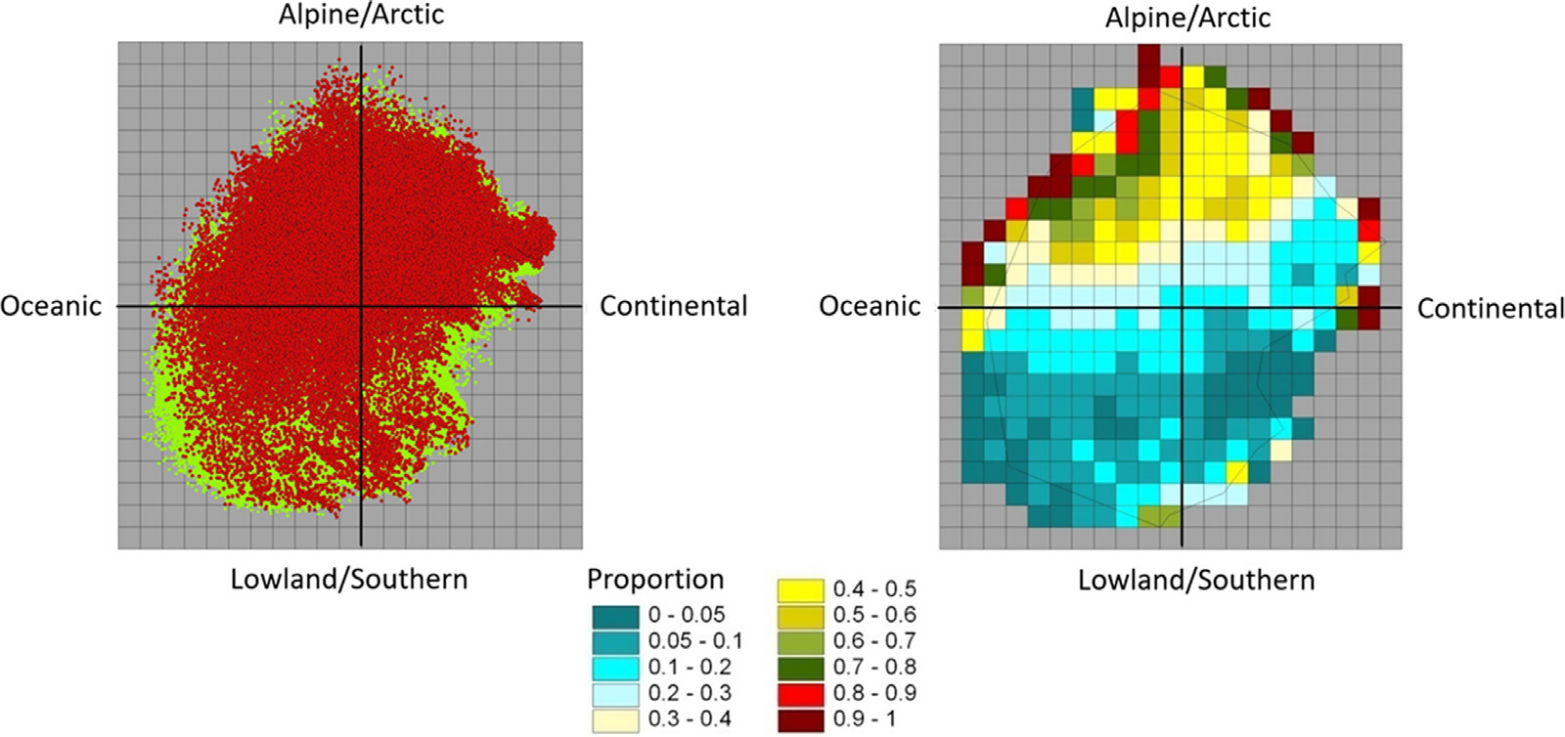
Left: Plots in the PCA-diagram of Norway (green dots) which are represented by protected areas (red dots. Right: the proportion of sample plots in protected areas extracted in a fishnet. The analysis reveals gaps along the coast and in the southern parts of Norway.

We have also tested the method for assessment of the representativeness of the localization of protected areas in Norway [6] (Fig. 5). The frequency representation offer an opportunity to relatively easy analyzing statistically the validity of a variety environmental hypothesis, although not pursued by us at the present stage.

In our analysis of cumulative effects by hydropower development in Nordland county in Norway [5], we collected spatial data representing modelled river gorges (the VEC) as well as existing infrastructure and hydroelectric development. We used these attributes to gather information about the position of a modelled valued ecological component (the midpoint of river gorges) and overlay these with the position of existing infrastructure, existing hydropower facilities, plans for future hydropower development and a map showing potential small-scale hydropower development. We collected their PCA position and then derived what proportion of river gorges in any given climatic segment either are presently affected by existing infrastructure and hydroelectric development, or would be affected by either proposed hydroelectric projects or other identified potential sites.

## Methodological aspects: the influence of scale and variables selected

The method may be vulnerable to certain effects related to scale, grid cell size and number of sample units. We specifically would like to address some issues and share some thoughts with respect to how to handle them in practical work.

• The spatial resolution of available climatic data usually restrict the users from addressing grain sizes below 1 km. However, our experience of the effect of grain sizes in the range 1–10 km, reveals consistent results over this entire range: in Bakkestuen et al. [3] the same four gradients were recovered in all models and in the same order. This shows that the potential aggregation modifiable areal unit problem (MAUP) scale problem [9,13] is not present within this range of grain sizes and this set of variables.
• We also have experienced that if the number of sample plots is high enough to represent the major directions of variation properly, which for Norway is achieved by 4000 cells (data points), a further increase in the size of the data set (increase the geographical resolution) has a minor impact on the overall results, except that the fraction of explained variation decreases slightly.
• The method presented is based on interpretation and analysis on a two-dimensional PCA output plot. It is possible to expand the method to include one or more extra dimensions by including more principal axes in the steps described.

## Conflict of interest

The Authors confirm that there are no conflicts of interest.
